# Prognostic prediction of subjective cognitive decline in major depressive disorder based on immune biomarkers: a prospective observational study

**DOI:** 10.1186/s12888-022-04513-x

**Published:** 2023-01-19

**Authors:** Meiti Wang, Zheyi Wei, Qinte Huang, Weijie Yang, Chenglin Wu, Tongdan Cao, Jie Zhao, Dongbin Lyu, Fan Wang, Ni Zhou, Haijing Huang, Mengke Zhang, Yiming Chen, Yi Xu, Weiliang Ma, Zheng Chen, Wu Hong

**Affiliations:** 1 Shanghai Mental Health Center, Shanghai Jiao Tong University School of Medicine, Shanghai, 200030 China; 2 Shanghai Pudong New Area Mental Health Center, School of Medicine, Tongji University, Shanghai, 200120 China; 3 Shanghai Huangpu District Mental Health Center, Shanghai, 200040 China; 4 Shanghai Key Laboratory of Psychotic Disorders, Shanghai, 201108 China

**Keywords:** Major depressive disorder, Subjective cognitive decline, Immune biomarkers, Predictive model

## Abstract

**Objective:**

Subjective cognitive decline (SCD) is highlighted in patients with major depressive disorder (MDD), which impairs objective cognitive performance and worsens the clinical outcomes. Immune dysregulation is supposed to be the potential mechanism of cognitive impairment. However, the peripheral immune biomarkers in patients troubled with MDD and SCD are not conventionally described.

**Methods:**

A prospective-observational study was conducted for 8 weeks. Subjective cognitive function was measured using the Chinese version of the 20-item perceived deficits questionnaire-depression (PDQ-D) and depression symptoms were evaluated with Hamilton Depression Rating Scale-17 (HDRS-17). Luminex assays were used to measure 48 immune cytokines in plasma at baseline. Integrating these results and clinicopathological features, a logistic regression model was used to develop a prognostic prediction.

**Results:**

Totally, 114 patients were enrolled in this study. Among the patients who completed follow-up, 56% (*N* = 50) had residual subjective cognitive decline, and 44% (*N* = 50) did not. The plasma levels of FGF basic, INF-γ, IL-1β, MCP-1, M-CSF and SCF were increased and the levels of IL-9, RANTES and PDGF-BB were decreased in the SCD group. Additionally, Basic FGF, IFN-γ, IL-1β, and SCF were positively correlated and IL-9, RANTES, and PDGF-BB were negatively correlated with the PDQ-D scores after treatment. Notably, combinations of cytokines (SCF and PDGF-BB) and PDQ-D scores at baseline showed good performance (The area under the receiver operating characteristic curve = 0.818) in the prediction of subjective cognitive decline.

**Conclusion:**

A prognostic model based on protein concentrations of SCF, PDGF-BB, and scores of PDQ-D showed considerable accuracy in predicting residual subjective cognitive decline in depression.

**Supplementary Information:**

The online version contains supplementary material available at 10.1186/s12888-022-04513-x.

## Introduction

Major depressive disorder (MDD) is a worldwide psychiatric condition associated with high recurrence rates and disability causes great financial losses [1]. The World Health Organization (WHO) reported that nearly 322 million people (estimated to be 4.4%) living with depression and the total rate increased by 18.4% between 2005 and 2015 [2]. While in China, MDD is the most prevalent mood disorder reported in 2019, and the lifetime prevalence and the 12-month prevalence of MDD are 3.4 and 2.1% respectively [3]. Despite the typical symptoms of MDD are depressed mood, diminished interests, and vegetative symptoms, accumulating evidence shows that cognitive impairment is one of the core symptoms of MDD [4, 5]. It is reported that cognitive problems persist 85–94% of the time during depressive episodes and 39–44% of the time during remissions [6]. And both in "The International Classification of Diseases-10" (ICD-10) and "The Diagnostic and Statistical Manual of Mental Disorders-5" (DSM-5), cognitive dysfunction is defined as one of the diagnostic criteria [7]. The symptoms of cognitive impairment contain memory decline, executive dysfunction, and decision-making difficulties both subjectively and objectively reported [8]. Subjective cognitive decline (SCD) reported by patients themselves, called cognitive symptoms, is more prominently correlated with depressive symptoms and clinical outcomes than objective cognitive impairment that represents cognitive performance [9]. Specifically, approximately half of the objectively remitted patients are not in subjective remission, and residual subjective cognitive problems such as memory or concentration trouble in 70% of outpatients with MDD [10], which contributes to an underestimation of the cognitive function causing worsen performance on cognitive tests [11]. Furthermore, evidence hypothesis that subjective function can represent the pre-morbid cognitive function [12], hence, subjective cognitive decline has been pointed to be a significant factor that causes poor socio-occupational ability and productivity [13].

Numerous studies have confirmed that immune dysfunction plays a key role in the pathological process of mental diseases such as schizophrenia, Alzheimer's disease(AD), bipolar disorder, and major depressive disorder [14–17]. One of the hypotheses is that peripheral immune marker activation damages the blood–brain system (BBB) which causes neuroinflammation and then exacerbates the pathogenic processes of depression [18]. On the other hand, neuroinflammation directly affects the microstructure of the hippocampus, thereby reducing the brain-derived neurotrophic factor (BDNF) level and signaling, resulting in diminished dendritic arborization, and leading to cognitive dysfunction in patients with MDD [19]. Existing evidence shows that plasma levels of interleukin-1β (IL-1β), a pro-inflammatory cytokine, are negatively associated with overall cognitive function in MDD patients [20], and interleukin-1Ra (IL-1Ra) is negatively associated with visual cognition, memory, and executive function [21]. Both the tumor necrosis factors (TNF-α) and the soluble tumor necrosis factor receptor 1 (sTNF-R1) are negatively correlated with impaired verbal memory, and elevated expression of tumor necrosis factor receptor superfamily member 1A (TNFRSF1A) and tumor necrosis factor receptor superfamily member 1B (TNFRSF1B) are negatively correlated with cognitive performance including working memory, executive functions, attention, auditory-verbal memory, the effectiveness of learning processes, and verbal fluency [22]. Besides the pro-inflammatory cytokines, studies also prove that chemokines and growth factors participate in the mechanism of linking depression and cognitive impairment, such as interleukin-8 (IL-8) is positively correlated with verbal fluency and eosinophil chemotactic protein 2 (eotaxin-2) is positive with generation ability and letter memory task [23]. Another reflected in a meta-analysis, the level of insulin-like growth factor can represent the severity of depression symptoms and cognitive disturbance [24]. Those studies demonstrate that not only the pro-inflammatory cytokines but also the chemokines and growth factors play a significant role in patients suffering from depression and cognitive impairment.

So far, most of the existing studies focused on the objective cognitive function in patients with MDD, and substantial evidence confirmed immune dysfunction as a cause of impaired cognitive performance in depression. However, fewer study to examine the potential relation between immune markers and residual subjective cognitive problems. Furthermore, the existing studies gave more attention to the relationship between pro-inflammatory cytokines such as IL-1β, and TNF-α in patients with MDD, while the correlation between immune markers was more complex, including chemokines and growth factors. Therefore, we selected a multi-cytokine array for simultaneous analysis to understand the role of inflammation in subjective cognitive decline in depression. And prognostic prediction analysis provides a strategy to further characterize the relationship between immune cytokines and residual subjective cognitive problems. Thus, this study aimed to find a predictive model of subjective cognitive impairment targeting immune cytokines in patients with depression. Firstly, all patients were tested for multiple cytokines, and then potential correlations between clinical parameters of immune cytokines were analyzed. Finally, a logistic regression model including clinical questionary and plasma immune cytokines was applied to predict the subjective cognitive function in MDD. This study not only provides a comprehensive understanding of the relationship between inflammation-related cytokines and subjective cognitive symptoms but also identifies new markers that predict subjective cognitive function in MDD.

## Methods

### Subjects and study procedure

**Fig. 1:**
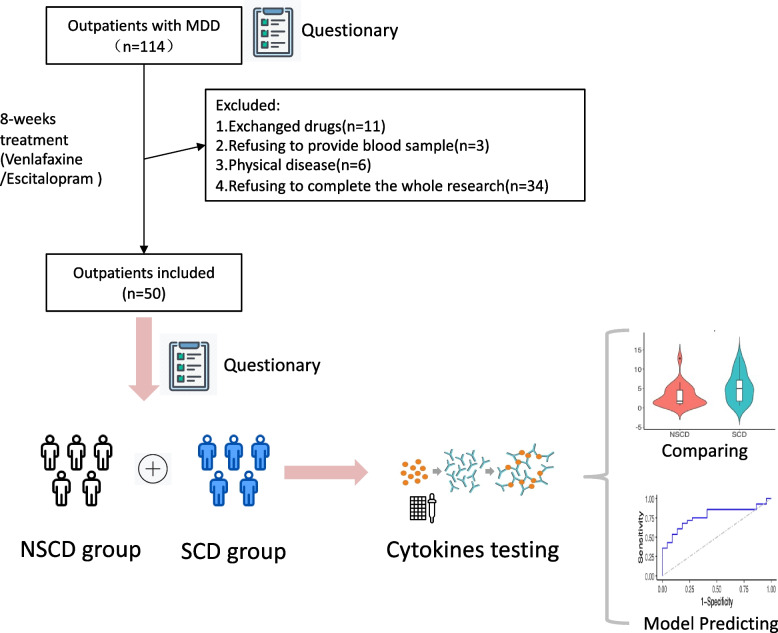
The study design and recruiting procedure. A total of 48 cytokines were selected for comparison between groups. Receiver operating characteristic (ROC) analysis was used to measure the accuracy of the model. Abbreviation: SCD: subjective cognitive decline; NSCD: non-subjective cognitive decline

The study design and the recruiting procedure were described in Fig. 1. All patients were recruited from the outpatient of Shanghai Mental Health Center in China from 2019 to 2020. Primarily, a total of one hundred and forty-four outpatients with depressive symptoms and subjective cognitive decline were recommended to participate in the research. Of these patients, thirty patients receiving benzodiazepines or antipsychotics were excluded, and one hundred and fourteen patients were finally included in the follow-up. Eleven patients who changed the drug from SSRI to SNRI were excluded from the study. Three patients refused to provide blood samples and sixteen patients who had the physical disease after recruiting in the study were ruled out. Ultimately, thirty-four patients who refused to complete the whole follow-up were excluded and fifty patients (male/female = 11/39) were enrolled. Either Escitalopram(10 mg-20 mg) or Venlafaxine(80 mg-225 mg) was prescribed by the doctor depending on the patient’s condition for 8 weeks.

All patients were required to meet the inclusion criteria:1) DSM-5 diagnosis criteria for depression and were assessed by an attending psychiatrist and above; 2) Han Chinese, aged 18–60 years; 3) was able to complete the entire inspection. 4) the score of Hamilton Depression Rating Scale-17 (HDRS-17) ≥ 17 and perceived deficits questionnaire-depression (PDQ-D) ≥ 21. 5) willing to participate. Exclusion criteria were: 1) pregnancy, lactation, or breastfeeding; 2) other mental illnesses, including schizophrenia, schizoaffective psychosis, bipolar disorder, substance abuse (alcohol/drugs); 3) major physical abnormalities like central nervous system disease, autoimmune disorders, acute infections, cancer.

### Clinical measurement and data collection

The clinical demographic data were collected by using a clinical research data manual including age, gender, education, and other clinical information at baseline. Clinical questionnaire assessments were collected at baseline and 8-week follow-up.

The HDRS-17 [25] was used to assess patients’ depressive symptoms by two psychometricians who had received concurrent training. All the subjects were suffering moderate to severe depressive episodes based on the HDRS-17 scores.

Subjective cognitive function was examined by the 20-item Perceived Deficits Questionnaire-Depression (PDQ-D, Chinses Version) including three cognitive domains (planning/concentration PC, retrospective memory RM, prospective memory PM), yielding a possible score range of 0–80, and with higher scores reflecting more pronounced symptoms [26]. A total PDQ-D score ≥ 21 means patients who suffer subjective cognitive decline [27]. The patients were separated into two groups which were the subjective cognitive decline group(SCD)and the non-subjective cognitive decline group(NSCD)according to the PDQ-D scores at 8 weeks. Patients with a total score of PDQ-D ≥ 21 were divided into the SCD group, and PDQ-D < 21 were divided into the NSCD group.

Functional disability is reflected by the Sheehan Disability Scale (SDS), which ranges from 0 (unimpaired) to 30 (highly impaired) and consists of three items: work or school, social life, and family life.

### Biochemical analysis

To explore the potential mechanism by which patients exist cognitive impairment, Luminex liquid suspension chip detection was performed to compare the differential expression of 48 cytokines including pro-inflammatory, chemokines, and growth factors (Supplementary Table 1).

All peripheral blood samples were collected between 8:00 to 12:00 by using anticoagulant tubes and the plasma was isolated by centrifugation (3000 rpm for 15 min at 4℃). The separated plasma was deposited at -80 ℃ until being used. According to the manufacturer's instructions using the Bio-Plex MagPix System, the plasma concentration of the 48 cytokines were simultaneously measured by the Bio-Plex Pro Human Cytokine Screening 48-Plex Panel (Bio-Rad, Hercules, CA, USA), and streamlined data were analyzed with the Bio-Plex Manager (Luminex, Austin, TX, USA), agented by Wayen Biotechnologies (Shanghai, China). The calculation of the concentration was based on the cytokine’s fluorescence values which resulted from the standard substance included in the 96-well plate. The concentration below or above the limit of the standard concentration could not be detected and treated as missing data. All the results are presented as picograms per milliliter.

### Statistical analysis

The Kolmogorov–Smirnov one-sample test was used to test the normal distribution of all data at first. Demographic and clinical variables of two groups were compared by t-test for normal distribution and Mann–Whitney U test for non-normal distribution, and categorical variables were analyzed by Chi-square test. Correlations between cytokines and clinical characteristics were examined by Pearson’s correlation coefficients or Spearman’s rank correlation coefficients. A binary logistics regression was applied to value the effects of which inflammatory markers were significantly associated with subjective cognitive impairment. The area under the receiver operating characteristic curve (AUROC), a parameter with a CI of 95%, was adopted to measure the accuracy of the model in predicting patients with or without subjective cognitive impairments. All statistical analyses and figures were carried out by using R software (Vienna, Austria, version 4.0.2). All *P* values were defined as a statistically significant set at 0.05.

## Results

### Clinical characteristics and subjective functional outcomes

The overall clinical characteristics of patients with MDD were shown in Table 1. There were twenty-nine female patients and eleven male patients with a mean age of 27.31 ± 7.21 years and a mean education level of 15.04 years. The baseline HRSD-17 score was 23.02 ± 4.77 and the average percentage of the reductive ratio was 58.75%. Twenty-eight patients with residual subjective cognitive decline were distributed into the SCD group and twenty-two patients into the NSCD group.

**Table 1:** The clinical characteristic of the total subjects enrolled

Variables	Total(*n* = 50)	
Age	Mean (SD)	27.31(7.21)
Gender	Male, n (%)	11(22%)
	Female, n (%)	39(78%)
Education level	Mean (SD)	15.04(3.02)
Marital status	Single, n (%)	27(54%)
	Married, n (%)	20(40%)
	Divorced, n (%)	3(6%)
Employment status	Employed, n (%)	32(64%)
	Unemployed, n (%)	18(36%)
Smoking	Yes, n (%)	12(24%)
	No, n (%)	38(76%)
Drinking	Yes, n (%)	16(32%)
	No, n (%)	34(68%)
Height	Mean (SD)	164.54(7.77)
Weight	Mean (SD)	56.38(11.29)
BMI	Mean (SD)	23.87(7.23)
Age of MDD onset (years)	Mean (SD)	23.88(7.23)
History of MDD episode	First episode, n (%)	25(50%)
	Recurrent episode, n (%)	25(50%)
Total course, month	Mean (SD)	31.38(36.09)
Baseline HRSD17 score	Mean (SD)	23.02(4.77)
Antidepressant drugs (ES/VF)	ES, n (%)	27(54%)
	VL, n (%)	23(46%)
HRSD-17 total score reduction, %	Mean (SD)	58.75(22.94)
PDQ-D score at baseline	Mean (SD)	36.94(12.80)
PDQ-D score at 8 weeks	Mean (SD)	31.44(20.96)
SDS score at baseline	Mean (SD)	14.24(7.00)
SDS score at 8 weeks	Mean (SD)	10.58(8.18)

*Abbreviation*: *MDD* Major depressive disorder, *BMI* Body mass index, *HRSD* the Hamilton Depression Rating Scale-17, *ES* Escitalopram, *VF* Venlafaxine, *PDQ-D* the Perceived Deficits Questionnaire-depression, *SDS* the Sheehan Disability Scale

Participants’ characteristics by group were reported in Table 2, and there were no significant differences in age, gender, education, height, weight, BMI, drinkers, smokers, duration of illness, and the age at disease onset between the two groups (all *P* > 0.05). The rate of HAMD reduction was greater than 50% in both groups but was significantly higher in the NSCD group than in the SCD group (*P* = 0.015). Patients in the SCD group were troubled with more subjective cognitive problems at baseline, and PDQ-D scores and three subitems differed significantly between the two groups (all *P* < 0.05). Excluding family life at baseline (*P* = 0.315), patients with residual subjective cognitive decline had reduced functional ability at baseline and 8-week, as reflected by the Sheen Disability Scale (all *P* < 0.05) (supplementary Table 2).

**Table 2:** The group analyses of the clinical characteristic according to assessment

		SCD (*n* = 28)	NSCD (*n* = 22)	*P* value
Age	Mean (SD)	28.25 ± 8.195	26.05 ± 5.59	0.295
Gender	Male, n (%)	7	4	
	Female, n (%)	21	18	
Education level	Mean (SD)	15.07 ± 3.615	15 ± 2.12	0.934
Marital status	Single, n (%)	15	12	0.928
	Married, n (%)	11	9	
	Divorced, n (%)	2	1	
Employment status	Employed, n (%)	21	11	0.83
	Unemployed, n (%)	7	11	
Smoking	Yes, n (%)	24	14	0.07
	No, n (%)	4	8	
Drinking	Yes, n (%)	6	10	0.071
	No, n (%)	22	12	
Height	Mean (SD)	164.69 ± 7.79	164.35 ± 7.94	0.832
Weight	Mean (SD)	56.75 ± 10.62	55.9 ± 12.37	0.649
BMI	Mean (SD)	24.44 ± 8.62	23.14 ± 5.03	0.739
Age of MDD onset (years)	Mean (SD)	24.44 ± 8.62	23.13 ± 5.03	0.739
History of MDD episode	First episode, n (%)	15	10	0.569
	Recurrent episode, n (%)	13	12	
Total course, month	Mean (SD)	26.56 ± 33.97	37.15 ± 38.56	0.338
HRSD17 at baseline	Mean (SD)	23.68 ± 4.86	22.18 ± 4.62	0.275
HRSD17 total score reduction, %	Mean (SD)	51.87 ± 22.62	67.5 ± 20.65	0.015^*^
Medicine (ES/VL)	ES, n (%)	12	15	0.075
	VL, n (%)	16	7	
PDQ-D-baseline	Mean (SD)	41.11 ± 15.01	29.23 ± 12.33	0.006^**^
PDQ-PC	Mean (SD)	18.03 ± 5.76	13.18 ± 5.93	0.005^**^
PDQ-RM	Mean (SD)	16.2 ± 7.04	12.36 ± 5.49	0.041^*^
PDQ-PM	Mean (SD)	6.91 ± 3.63	3.86 ± 2.55	0.003^**^
PDQ-D-Week 8	Mean (SD)	46.21 ± 15.32	12.62 ± 7.89	0.001^**^
PDQ-PC	Mean (SD)	18.14 ± 5.71	5.27 ± 3.87	0.001^**^
PDQ-RM	Mean (SD)	20.18 ± 7.55	6.36 ± 4.16	0.001^**^
PDQ-PM	Mean (SD)	7.89 ± 3.33	1.00 ± 1.20	0.001^**^

*Abbreviation*: *SCD* Subjective cognitive decline, *NSCD* Non-subjective cognitive decline, *BMI* Body mass Index, *HRSD* the Hamilton Depression Rating Scale-17, *ES* Escitalopram, *VF* Venlafaxine, *PDQ-D* the Perceived Deficits Questionnaire-depression, *PC* Planning/concentration, *RM* Retrospective memory, *PM* Prospective memory

^*^ Significantly difference (*p* < 0.05)

^**^ Significantly difference (*p* < 0.01)

### Comparison of immune cytokine protein levels

**Fig. 2:**
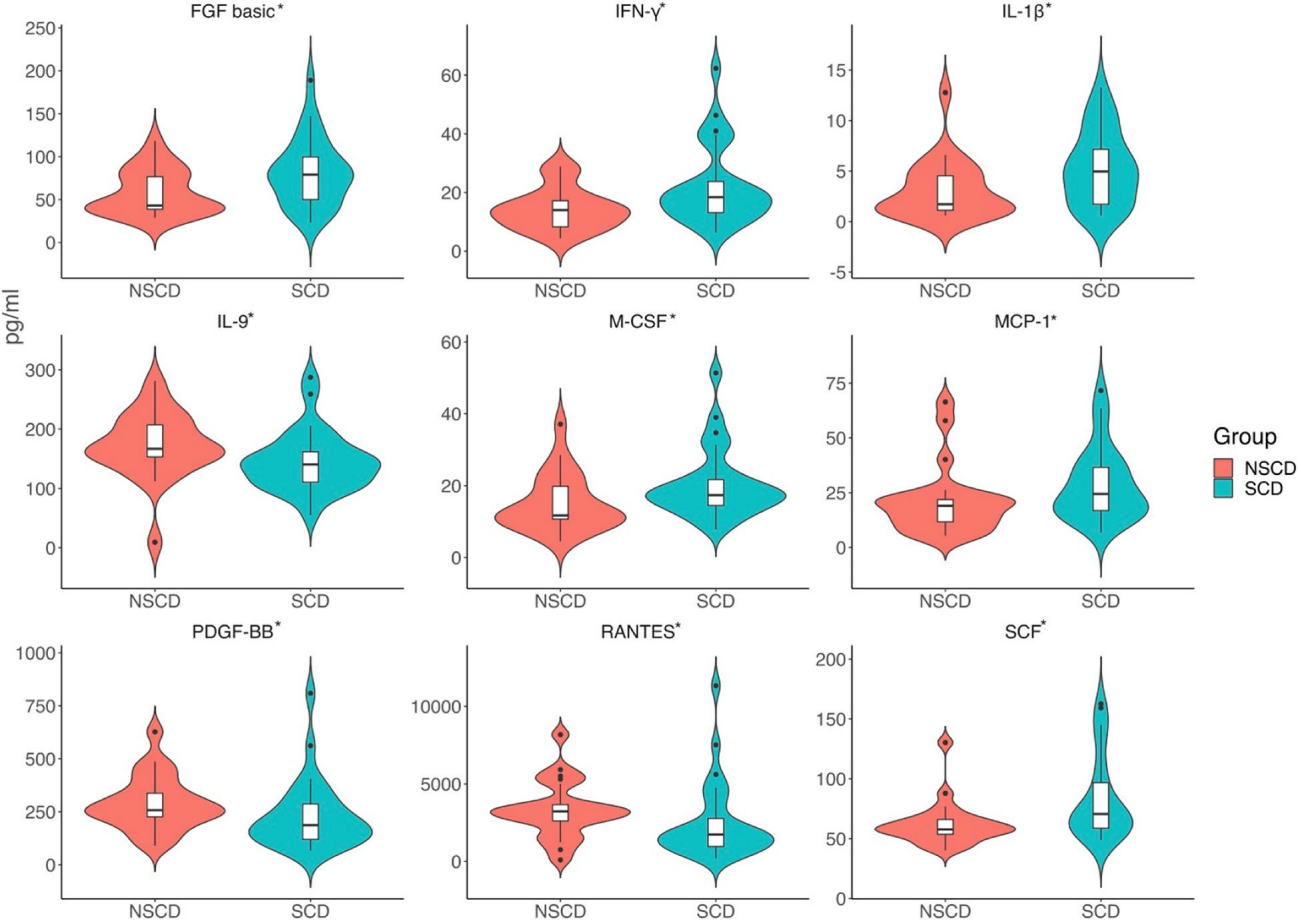
Difference of the cytokines level between the NSCD and SCD group at baseline. Violin plots were used to visualize the distribution of numerical data and depicted summary statistics and the density of each variable. Abbreviation: pg/ml: picograms per milliliter; FGF basic: basic fibroblast factor; INF-γ: interferon gamma; IL-1β: interleukin-1 beta; SCF: stem cell factor; MCP-1: monocytechemoattractantprotein-1; M-CSF: macrophage colony-stimulating factor; IL-9: interleukin-9; PDGF-BB: platelet-derived growth factor BB; RANTES: regulated on activation, normal T cell expressed and secreted. *Significantly difference (*p* < 0.05)

Cytokines including IFNa2, GM-CSF, IL-1ra, IL-2, IL-5, IL-6, IL-15, MIF, IL-12, and VEGF were excluded because the missing data for these cytokines were greater than 30%. As shown in Fig. 2, the protein concentrations of FGF basic, INF-γ, IL-1β, SCF, MCP-1, and M-CSF were increased in the SCD group, while IL-9, PDGF-BB, and RANTES were decreased (all *P* < 0.05). No difference was found in other cytokines (Supplementary Table 3).

### Correlations of peripheral immune cytokine with subjective cognitive function

**Fig. 3:**
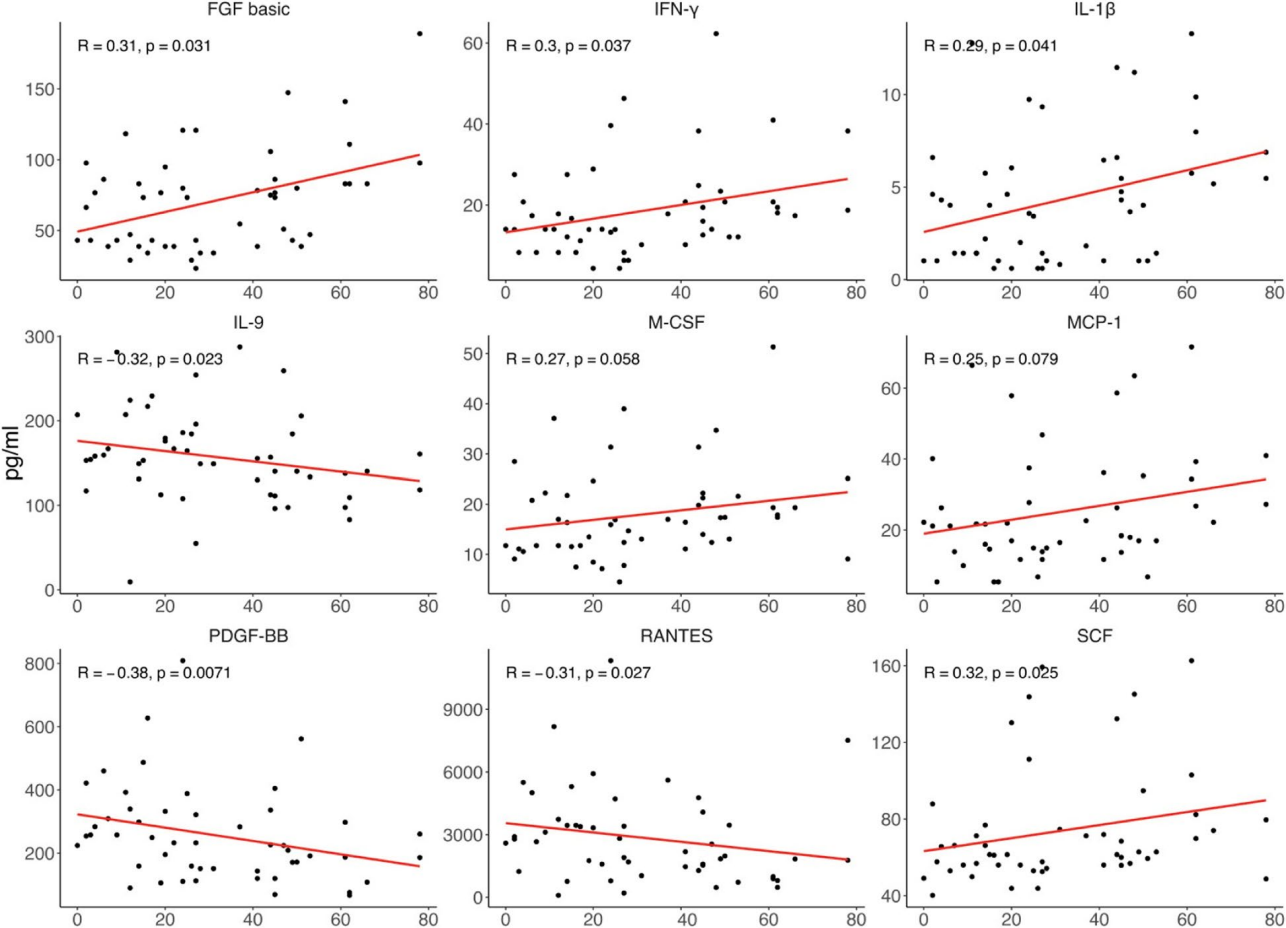
Relationship between cytokines at baseline and the PDQ-D scores at week 8. Abbreviation: FGF basic: basic fibroblast factor; INF-γ: interferon gamma; IL-1β: interleukin-1 beta; SCF: stem cell factor; MCP-1: monocytechemoattractantprotein-1; M-CSF: macrophage colony-stimulating factor; IL-9: interleukin-9; PDGF-BB: platelet-derived growth factor BB; RANTES: regulated on activation, normal T cell expressed and secreted. Perceived PDQ-D: perceived deficits questionnaire-depression

Among the nine cytokines differed between the two groups, the PDQ-D scores at week 8 were positively correlated with the protein concentrations of FGF basic(R = 0.31; *P* = 0.031), IFN-γ(R = 0.3; *P* = 0.037), IL-1β(R = 0.29; *P* = 0.041), and SCF(R = 0.32; *P* = 0.025), and negatively associated with the levels of IL-9(R = -0.32; *P* = 0.023), RANTES(R = -0.31; *P* = 0.027), and PDGF-BB(R = -0.38; *P* = 0.0071) (Fig. 3). For further analysis, no correlation was observed between protein concentrations of the nine cytokines and baseline PDQ-D scores (Supplementary Fig. 1). In addition, the correlations between the nine cytokines and the PDQ-D subitems and SDS subitems were shown in Supplementary Fig. 2.

### Predictive model for the decline in subjective cognitive function

**Fig. 4:**
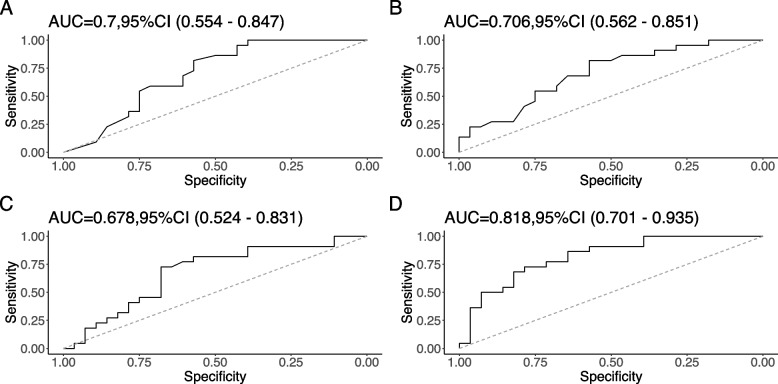
Prognostic prediction of residual subjective cognitive decline. **A** ROC curves for PDQ-D scores at baseline; **B** ROC curves for SCF level at baseline; **C** ROC curves for PDGF-BB level at baseline; **D** ROC curves for the combination (PDQ-D scores + SCF + PDGF-BB) at baseline to predict the subjective cognitive function. Abbreviation: area under the receiver operating characteristic curve (AUC)

The results of the ROC analysis assessing cytokine concentrations to predict residual subjective cognitive function were shown in Fig. 4. The levels of SCF and PDGF-BB showed significant value for predicting subjective cognitive decline, with an AUROC of 0.7 (95%CI = 0.933–0.993) and 0.678 (95%CI = 1.000–1.009). Furthermore, the severity of subjective cognitive function at baseline had a significant effect on cognitive prognosis. Scores of PDQ-D at baseline and levels of SCF and PDGF-BB combination showed a predictive value of AUROC of 0.818(CI = 0.701–0.935).

## Discussion

The prominent finding of this study was that peripheral immune cytokines were associated with the residual subjective cognitive function in MDD after 8 weeks of treatment, independent of pre-treatment subjective cognitive function. This finding demonstrated that SCF and PDGF-BB and the combination of the two growth factors with PDQ-D scores at baseline could predict the subjective cognitive function after treatment in MDD. These results robustly implicated the relationship between immune dysregulation and residual subjective cognitive function in depression.

The first finding of this study suggests that subjective cognitive problems troubled both symptomatic and remitted depressed patients. These results were consistent with previous studies that demonstrated cognitive problems are key clinical factors of functional disability [28, 29]. One hypothesis is that the decrease in self-evaluation and the increase in self-doubt and negative self-evaluation are the main reasons why patients are more sensitive to the subtle deterioration in cognitive function and underestimate their cognitive ability [30]. In addition, the observed subjective cognitive decline is associated with greater socio-occupational difficulties and functional disabilities [31, 32], which was shown in this study reflected by SDS scores. Interestingly, both the recurring depressive episodes and the longer duration of illness can deteriorate the subjective cognitive function [32], but no significant difference was found in this study.

The second finding of this study was that depressed patients with residual subjective cognitive decline had differences in immune cytokines prior to treatment. It should be noted that the results were partially consistent with previous studies focusing on objective cognitive function and increased plasma inflammatory biomarkers IL-1β [33, 34] and MCP-1 [35]. And another machine learning analysis shows that plasma SCF contributes significantly to differentiate cognitive impairment in patients with late-life depression [36], which is also consistent with the results of this study. Furthermore, this study found that PDGF-BB decreased in patients with SCD, which was consistent with previous studies on mild cognitive impairment, but inconsistent with studies on AD [37]. The reason for this discrepancy may be that MCI patients predominantly report subjective cognitive symptoms, whereas AD occupies more objective cognitive symptoms. In contrast to the previous study focusing on depressive symptoms, the present study found a significant increase in IFN-γ levels and a significant decrease in IL-9 levels in the SCD group with more severe depressive symptoms [38, 39]. Differences in the clinical characteristics of patients selected for enrollment in those studies explain the inconsistent results. In addition, it has been reported that hippocampal FGF basic mRNA density is reduced in the postmortem brain of individuals suffering from major depressive disorder [40]. While in this study, we found that the peripheral levels of FGF basic were increased in the SCD group which implied the different mechanisms underline the peripheral and central immune systems.

The third finding of this study was that FGF basic, IFN-γ, IL-1β, and SCF were negatively associated with residual subjective cognitive function, whereas IL-9, PDGF-BB, and RANTES were positively associated with residual subjective cognitive function. Firstly, the results indicated a strong link between subjective cognitive problems and abnormalities in immune mechanisms involving chronic adaptive immunity. In the preclinical study, increased IL-1β has adverse effects on memory and cognitive function and negatively influences the proliferation, differentiation, and survival of hippocampal NPCs in both juvenile and adult animal models [41]. Particularly, IL-1β produced by microglia also promotes the Th-1 responses to produce IFN-γ, which inhibits IL-9 secretion and promotes neuroinflammation [42]. These correlations between IL-1β, IFN-γ, and IL-9 explain the negative association of IFN-γ and IL-1β with subjective cognitive function and the positive association of IL-9 with subjective cognitive function in this study. Furthermore, IL-1β interacts with RANTES and registers an important role in brain pathology [43], but a previous study found no correlation between RANTES and objective cognitive function [44]. In this study, a positive correlation was observed between RANTES and subjective cognitive function, and the mechanism of this correlation needs further investigation. Secondly, evidence strongly suggested that another mechanism was related to the BBB, which connected the peripheral and central immune systems. PDGF-BB, targeting the PGDFRβ + pericytes [45], cooperates with FGF basic in maintaining the integrity and stabilization of BBB, enhancing the function of endothelial cells and the compactness of the cells [46]. The imbalance between those two cytokines could deteriorate the BBB completion and accelerate the other pro-inflammatory cytokines cross the BBB.

The fourth finding of this study was that SCF and PDGF-BB predicted residual subjective cognitive function in depressed patients, respectively. The mechanism was point to the interaction between different neuroprotectors. SCF is a neurotrophic factor which contributes to neuroprotective and immune effects that are involved in the process of remission/recovery from depression [47]. SCF can down regulate microglial expression of the inflammation-associated cytokines, such as IL-1β and enhance microglial expression of the mRNAs of nerve growth factor (NGF) and BDNF. PDGF-BB has been reported to protect the neurons from oxidative insults and energy deprivation by upregulating the PDGFR-β signaling, targeting the NR2B receptors and the PI3K/AKT pathway [48]. Moreover, the exogenous administration of PDGF-BB has been demonstrated to prevent glutamate-induced hippocampal neuronal injury and death [49]. Besides those studies, evidence also prove that the protection function of serotonin receptor agonist treatment to the NMDA-induced cell death was mediated through increased expression of PDGFR-β in primary hippocampal neurons [49]. But until to now, the interaction between SCF and PDGF-BB in subjective cognitive decline in MDD is still unclear and requires further study.

Notably, the findings also suggest that the combination of clinical markers and peripheral immune cytokines was more accurate and effective in predicting residual subjective cognitive decline in depressed patients. Interestingly, no correlations were found in the baseline PDQ-D scores and immune cytokines. Whether these results are related to medication use requires further study. In clinical, PDQ-D is convenient for doctors to evaluate the patient's subjective cognitive symptoms, while some patients exaggerate or conceal their symptoms, resulting in inaccurate measurements. However, objective biomarkers, which avoid subjective influences, can help clinicians predict residual subjective function and develop new interventions in the early stages of patient treatment. To our surprise, the findings highlight the role of growth factors as immune markers in subjective cognitive impairment in depression, and the involvement in neuroinflammation and peripheral immunity in depression deserves further attention. Next work needs to expand the sample size and follow-up time to further investigate the accuracy of the model.

Several limitations need to be highlighted in this study. First, we used a self-reported cognitive assessment questionnaire to assess subjective cognitive decline in patients with depression. Although the reliability and validity of the PDQ-D have been studied in Chinese, the shortcoming of this questionnaire is that it cannot represent the whole field of cognitive function. In addition, we did not measure objective cognitive function in this study, which covers a major part of cognitive function, and the absence of this part is one of the important flaws of the article. Second, because the sample/assay fluid is in a well, cytokines drawn into multiple screening plates have potential interactions with other antibodies. And multiplex testing techniques may underestimate the levels of some cytokines. All these reasons could affect our results. Third, only 50 patients in our study completed a complete follow-up, a number too small to be statistically biased. Our study is prospective with small sample size, and further research needs to be carried out in a larger sample size.

## Conclusion

In conclusion, depressed patients with residual cognitive impairment had alterations in their pretreatment pro-inflammatory cytokines, chemokines, and growth factors, suggesting an overall change in the inflammatory network. The clinical questionary combined with immune markers(SCF and PDGF-BB) efficiently predicts subjective cognitive decline after treatment. For patients with subjective cognitive decline, clinicians can explore new therapeutic directions by focusing on whether such patients have abnormal levels of immune inflammation. Also, a predictive method combining inflammatory factors and clinical indicators can expand clinical ideas for identifying new diagnostic and therapeutic approaches.

## Supplementary Information

**Additional file 1:** **Supplementary table 1.** The 48 cytokines tested included pro-inflammatory cytokines, chemokines, and growth factors. ABB: abbreviation. **Supplementary table 2.** Functional ability between the SCD group and NSCD group. **Supplementary table 3.** Difference of the cytokines level between the NSCD and SCD group at baseline. **Supplementary figure 1.** Correlation of the cytokines level and PDQ-D scores at baseline. **Supplementary figure 2.** Relationship between cytokines at baseline and the PDQ-D subtests (A), SDS scores and subtests (B) both at baseline and after 8 weeks treatment. 

## Data Availability

The original data can be acquired by connecting to the corresponding author.
