# Supplementary material for: Prognostic prediction of subjective cognitive decline in major depressive disorder based on immune biomarkers: a prospective observational study
Source: BMC Psychiatry. 2023 Jan 19;23:54. doi: 10.1186/s12888-022-04513-x (PMC9850523; doi:10.1186/s12888-022-04513-x)
Supplement: Supplementary file 1 — Additional file 1: Supplementary table 1. The 48 cytokines tested included pro-inflammatory cytokines, chemokines, and growth factors. ABB: abbreviation. Supplementary table 2. Functional ability between the SCD group and NSCD group. Supplementary table 3. Difference of the cytokines level between the NSCD and SCD group at baseline. Supplementary figure 1. Correlation of the cytokines level and PDQ-D scores at baseline. Supplementary figure 2. Relationship between cytokines at baseline and the PDQ-D subtests (A), SDS scores and subtests (B) both at baseline and after 8 weeks treatment. [file 12888_2022_4513_MOESM1_ESM.docx]

Supplementary table 1: The 48 cytokines tested included pro-inflammatory cytokines, chemokines, and growth factors. ABB: abbreviation

| ABB | Cytokines | ABB | Cytokines | ABB | Cytokines |
| --- | --- | --- | --- | --- | --- |
| IL-1α | Interleukin-1α | IL-15 | Interleukin-15 | GRO-α/CXCL1 | Growth-regulated oncogene-alpha/Chemokine (C-X-C motif) ligand 1 |
| IL-1β | Interleukin-1β | IL-16 | Interleukin-16 | MIG/CXCL9 | Monokine induced by gamma/Chemokine (C-X-C motif) ligand 9 |
| IL-1ra | Interleukin-1ra | IL-17A | Interleukin-17A | IP10/CXCL10 | IFN-gamma-inducible protein 10/Chemokine (C-X-C motif) ligand 10 |
| IL-2 | Interleukin-2 | IL-18 | Interleukin-18 | SDF-1α/CXCL12 | Stromal cell-derived factor 1/Chemokine (C-X-C motif) ligand 12 |
| IL-2Rα | Interleukin-2Rα | TNF-α | Tumor necrosis factors -α | TRAIL | TNF-related apoptosis-inducing ligand |
| IL-3 | Interleukin-3 | TNF-β | Tumor necrosis factors -β | HGF | Hepatocyte growth factor |
| IL-4 | Interleukin-4 | IFN-α2 | Human interferon alpha-2 | SCGF-β | Stem cell growth factor-β |
| IL-5 | Interleukin-5 | IFN-γ | Interferon-γ | M-CSF | Macrophage colony-stimulating factor |
| IL-6 | Interleukin-6 | MIF | Macrophage migration inhibitory factor | G-CSF | Granulocyte colony-stimulating factor |
| IL-7 | Interleukin-7 | MCP-1/CCL2 | Monocyte chemoattractant protein-1/Chemokine (CC-motif) ligand 2 | GM-CSF | Granulocyte-macrophage colony-stimulating factor |
| IL8/CXCL8 | Interleukin-8/Chemokine (C-X-C motif) ligand 8 | MIP-1α/CCL3 | Macrophage inflammatory protein-1α/Chemokine (CC-motif) ligand 3 | FGF basic | fibroblast growth factor basic |
| IL-9 | Interleukin-9 | MIP-1β/CCL4 | Macrophage inflammatory protein-1β/Chemokine (CC-motif) ligand 4 | PDGF-BB | Platelet-derived growth factor-BB |
| IL-10 | Interleukin-10 | RANTES/CCL5 | Regulated on activation, normal T cell expressed and secreted/Chemokine (CC-motif) ligand 5 | SCF | Stem cell factor |
| IL-12(p40) | Interleukin-12(p40) | MCP-3/CCL7 | Monocyte chemoattractant protein-3/Chemokine (CC-motif) ligand 7 | VEGF-A | Vascular endothelial growth factor A |
| IL-12 (p70) | Interleukin-12(p70) | Eotaxin/CCL11 | Eosinophil chemotactic protein 2/Chemokine (CC-motif) ligand 11 | β-NGF | β-Nerve Growth Factor |
| IL-13 | Interleukin-13 | CTACK/CCL27 | Cutaneous T cell-attracting chemokine/Chemokine (CC-motif) ligand 27 | LIF | Leukemia inhibitory factor |

Supplementary table2: Functional ability between the SCD group and NSCD group

| Sheehan Disability Scale |  | SCD (n=28) | NSCD (n=22) | P value |
| --- | --- | --- | --- | --- |
| Family life | Mean (SD) |  |  |  |
| baseline |  | 5.00 ±2.55 | 4.23 ±2.81 | 0.315 |
| week 8 |  | 4.57 ±2.73 | 1.55 ±1.82 | <0.001^**^ |
| Work or school | Mean (SD) |  |  |  |
| baseline |  | 5.93 ±2.34 | 3.86 ±2.27 | 0.003^**^ |
| week 8 |  | 5.46 ±2.89 | 2.05 ±1.94 | <0.001^**^ |
| Social life | Mean (SD) |  |  |  |
| baseline |  | 5.54 ±2.77 | 3.32 ±2.32 | 0.004^**^ |
| week 8 |  | 4.82 ±2.92 | 1.55 ±1.82 | <0.001^**^ |

Table3: Abbreviation: SCD: subjective cognitive decline; NSCD: non-subjective cognitive decline.

*Significantly difference (p < 0.05); ** Significantly difference (p < 0.01).

Supplementary table3: Difference of the cytokines level between the NSCD and SCD group at baseline.

|  | SCD (n=28) | NSCD (n=22) | p |
| --- | --- | --- | --- |
| CTACK | 412.12 (120.10) | 459.97 (156.47) | 0.242 |
| Eotaxin | 39.44 (12.98) | 44.06 (17.01) | 0.297 |
| G-CSF | 63.95 (50.65) | 85.27 (61.17) | 0.194 |
| GRO-α | 504.24 (182.77) | 518.76 (161.02) | 0.767 |
| HGF | 310.45 (125.66) | 366.04 (161.87) | 0.191 |
| IL-1α | 34.32 (21.73) | 41.38 (28.02) | 0.335 |
| IL-2Rα | 57.77 (37.62) | 75.83 (43.12) | 0.127 |
| IL-3 | 2.78 (1.62) | 2.32 (1.67) | 0.327 |
| IL-4 | 1.79 (1.50) | 2.72 (2.03) | 0.080 |
| IL-7 | 25.92 (14.55) | 33.95 (18.52) | 0.102 |
| IL-8 | 6.06 (7.80) | 8.92 (9.30) | 0.252 |
| IL-9 | 160.04 (43.20) | 149.91 (45.20) | 0.427 |
| IL-10 | 6.81 (7.84) | 9.44 (11.64) | 0.369 |
| IL-12(P40) | 132.03 (162.72) | 231.00 (184.49) | 0.053 |
| IL-12(P70) | 81.31 (297.84) | 11.75 (16.86) | 0.222 |
| IL-13 | 4.37 (5.40) | 7.44 (7.10) | 0.099 |
| IL-16 | 67.64 (36.23) | 83.66 (54.55) | 0.242 |
| IL-17 | 12.37 (14.12) | 15.74 (13.01) | 0.385 |
| IL-18 | 58.36 (28.83) | 62.42 (33.54) | 0.654 |
| IP-10 | 205.77 (80.24) | 245.80 (142.76) | 0.245 |
| LIF | 58.73 (34.95) | 79.51 (58.42) | 0.147 |
| MCP-3 | 3.50 (2.23) | 3.13 (2.58) | 0.595 |
| MIG | 104.14 (57.12) | 115.53 (75.73) | 0.560 |
| MIP-1α | 1.79 (1.27) | 2.27 (1.42) | 0.224 |
| MIP-1β | 144.01 (59.27) | 141.42 (49.24) | 0.867 |
| β-NGF | 5.28 (3.73) | 4.71 (3.66) | 0.588 |
| SCGF-β | 97226.65 (28236.68) | 91641.43 (36434.83) | 0.556 |
| SDF-1α | 789.81 (225.68) | 714.02 (196.63) | 0.211 |
| TNF-α | 64.64 (28.47) | 58.62 (26.44) | 0.444 |
| TNF-β | 248.18 (66.15) | 218.25 (74.85) | 0.146 |
| TRAIL | 58.65 (42.35) | 69.21 (44.73) | 0.401 |

Supplementary figure 1

Supplementary figure1：Correlation of the cytokines level and PDQ-D scores at baseline.

Supplementary figure2

Supplementary figure2: Relationship between cytokines at baseline and the PDQ-D subtests(A), SDS scores and subtests(B) both at baseline and after 8 weeks treatment.
